# Inducible ATF3–NFAT axis aggravates podocyte injury

**DOI:** 10.1007/s00109-017-1601-x

**Published:** 2017-10-16

**Authors:** Hong Zhang, Shun Liang, Yue Du, Ruizhao Li, Chaosheng He, Wenjian Wang, Shuangxin Liu, Zhiming Ye, Xinling Liang, Wei Shi, Bin Zhang

**Affiliations:** 1 Department of Nephrology, Guangdong General Hospital, Guangdong Academy of Medical Sciences, 106# Zhongshan No. 2 Road, Guangzhou, 510080 China; 2 Southern Medical University, Guangzhou, 510515 China; 3 Department of Nephrology, The Fifth Affiliated Hospital, Sun Yat-sen University, Zhuhai, 519000 China; 4 School of Medicine, South China University of Technology, Guangzhou, 510006 China

**Keywords:** Podocyte, Apoptosis, Injury, Activating transcription factor 3, Nuclear factor of activated T cell

## Abstract

**Abstract:**

Podocyte injury and loss contribute to proteinuria, glomerulosclerosis, and eventually kidney failure. Activating transcription factor 3 (ATF3) is a stress inducible transcription factor that is transiently expressed following stimulation. However, we show for the first time an induction of ATF3 in podocytes from patients with chronic kidney disease, including minimal change disease, focal segmental glomerulosclerosis, and diabetic nephropathy. The role of ATF3 induction in podocytes under chronic conditions is currently unknown. Compared with the control (C57 or BKS), ATF3 expression was elevated in animal model of proteinuria (LPS-treated C57 mice) and the model of diabetic nephropathy (db/db mice). Similarly, ATF3 was increased in high glucose (HG)-treated, lipopolysaccharide (LPS)-treated, or Ionomycin-treated podocytes in vitro. Overexpression of ATF3 increased podocyte apoptosis and decreased expression of podocin, the cell marker of podocyte; in contrast, ATF3–small interfering RNA knockdown reduced podocyte apoptosis and increased podocin expression. The translocation of ATF3 to the nucleus was increased upon stimulation. ATF3 directly modulates the regulation of NFATc1 gene promoter activity and alters the expression of Wnt6 and Fzd9, direct target genes of NFATc1 signaling. The ATF3 binding site of NFATc1 gene promoter is located at the region 671–775 base pairs upstream of the transcription start site. These results indicate a novel inducible axis of ATF3–NFAT in podocyte injury and loss.

**Key messages:**

• The stress factor ATF3 is induced in podocytes from proteinuric patients, including diabetes.

• ATF3 increased podocyte apoptosis and injury.

• ATF3 directly modulates the regulation of NFATc1 gene promoter activity.

## Introduction

Podocyte injury and loss contribute to proteinuria and progressive glomerulosclerosis [1–3]. The glomerular filtration barrier consists of podocytes, endothelium, and the intervening glomerular basement membrane [4]. By forming the only connection between adjacent podocytes, the slit diaphragm limits plasma protein leakage by acting as a size barrier, analogous to a sieve. Mutation and abnormalities in the slit diaphragm proteins in junctional domain (podocin, nephrin, CD2AP, and others) are associated with podocyte injury and loss, proteinuria, and consequently glomerulosclerosis [5–7]. Podocytes play such a key role in maintaining the structural and functional integrity of the filtration barrier that the progression of kidney disease always has podocyte involvement [8]. There is evidence that apoptosis contributes to podocyte loss [9]. In diabetic nephropathy, podocyte apoptosis is an early glomerular phenotype of progressive podocyte loss and proteinuria [10], which is induced by various injurious factors, including high glucose, TGF-β, and angiotensin II [9, 11–14].

Nuclear factor of activated T cell (NFAT) is a family of transcription factors originally identified as important mediators in cytokine gene expression during the immune response [15]. The inhibition of calcineurin-NFAT axis is the cornerstone of most immunosuppressive treatment. Clinically, the most commonly used inhibitor of calcineurin, cyclosporine A, directly reduces proteinuria in proteinuric patients with minimal change disease (MCD) [16, 17], focal segmental glomerulosclerosis (FSGS) [18, 19], or membranous nephropathy (MN) [20], indicating that calcineurin-NFAT activation may cause podocyte injury and proteinuria. Recently, multiple lines of evidence, including in vivo evidence, indicated that activation of NFAT in podocytes causes proteinuria and glomerulosclerosis [21–24]. NFAT proteins are primarily phosphorylated and found in the cytoplasm of resting cells. When calcineurin is activated, NFAT is dephosphorylated and then translocates into the nucleus and regulate transcription of NFAT-dependent genes. In podocytes, these direct transcriptional targets of NFATc1 signaling include wnt6, Fzd9, and urokinase-type plasminogen activator receptor (uPAR), through which NFAT exerts the injurious action on podocytes [21, 22].

Activating transcription factor 3 (ATF3) is a member of the ATF/cyclic adenosine monophosphate (cAMP)-responsive element-binding family of proteins that acts as a stress-inducible transcriptional factor, and ATF3 is induced by a wide range of stress stimuli and transiently expressed following stimulation [25]. Activated ATF3 can either homodimerize and repress transcription of various promoters with ATF sites [26] or heterodimerize with bZip proteins, c-Jun, Jun B, ATF2, or gadd153/CHOP10 (C/EBP homologous protein) and thus activate transcription of target genes [27].

In this study, we found an inducible expression of ATF3 in glomerular podocytes from proteinuric patients with minimal change disease (MCD), focal segmental glomerulosclerosis (FSGS), and diabetic nephropathy (DN). And we further demonstrated that ATF3 acted as a transcriptional activator in damaged podocytes, that ATF3 upregulates NFATc1 gene promoter activity via direct binding to the NFAT promoter, and thus aggravates podocyte injury and apoptosis. Inhibition of ATF3 induction prevented podocyte injury and apoptosis.

## Materials and methods

### Patients

The study of patients was conducted in accordance with the Second Helsinki Declaration and was approved by the Ethics Committee for Human Research of Guangdong General Hospital (no. GDREC2015227A). Kidney biopsy samples were obtained from proteinuric patients with minimal change disease (MCD), focal segmental glomerulosclerosis (FSGS), or diabetic nephropathy (DN), and normal kidney tissues were obtained from patients with renal cell carcinoma (adjacent normal tissues). Written informed consent for kidney samples for research purposes was obtained from patients at the time of biopsy or before operation.

### Animals and treatments

All animal studies were approved by the Ethics Committee for Animal Research of Guangdong General Hospital. Male C57BL/6 mice were purchased from Center of Laboratory Animal Science of Guangdong, China. For the murine model of transient proteinuria (lipopolysaccharide (LPS)-treated C57 mice), 200 μg LPS (L-2880, Sigma-Aldrich) in a total volume of 100 μl was intraperitoneally injected. C57BL/KsJ-db/db mice and age-matched wild-type (BKS) mice were purchased from Model Animal Research Center of Nanjing University and housed in the animal center of Sun Yat-sen University Zhongshan School of Medicine, Guangzhou, China. Mice were anesthetized (ketamine, 70 mg/kg, intraperitoneally injected), and kidney tissue was collected.

### Cell culture and treatments

The conditionally immortalized mouse podocyte cell line was kindly provided by Prof. Jochen Reiser (Rush University Medical Center, Chicago, IL, USA) and cultured as described previously [28]. Briefly, podocytes were cultured at 33 °C in RPMI-1640 medium (Gibco BRL, Gaithersburg, MD, USA) supplemented with 10% fetal bovine serum (FBS, Gibco BRL, Gaithersburg, MD, USA) and recombinant IFN-γ (growth permissive conditions; CYT-358, ProSpec, Tany Technogene Ltd., Ness Ziona, Israel). To induce differentiation, podocytes were reseeded and cultured at 37 °C in 50 cm^2^ culture dish coated with 12 mg/ml type-I collagen (BD Bioscience, Bedford, MA, USA) and in Dulbecco’s modified Eagle’s medium (with 5.3 mM glucose, Invitrogen, Carlsbad, CA, USA) supplemented with 5% FBS, deprived of IFN-γ (growth restrictive conditions) for 10–13 days. After differentiation, podocytes was confirmed by the identification of synaptopodin, a podocyte differentiation marker. Before stimulation, we made differentiated podocytes quiescent by serum-starvation overnight. And then we treated podocytes with HG (add to 30 mM glucose),LPS (100 μg/ml), Ionomycin (2 μM) or small interfering RNA (siRNA) (50 nM) for 1, 2, 4, 6, or 72 h.

### siRNA transfection and infection of adenovirus

The siRNA sequences targeted ATF3 and control–siRNA were designed and synthesized by RiboBio Co., Ltd. (Guangzhou, China). Transfection experiments were performed following transfection reagent, Lipofectamine 2000 (Invitrogen, Thermo Fisher Scientific, Waltham, MA) protocols. The sequences of ATF3–siRNAs used in this study were as follows: ATF3–siRNA no. 1, 5′CCTGACACCCTTTGTCAAG dTdT-3′; ATF3–siRNA no. 2, 5′ GCTGCCAAGTGTCGAAACA dTdT-3′; and ATF3–siRNA no. 3, 5′ CCTCTTTATCCAACAGATA dTdT-3′. To overexpress ATF3 in podocytes, the adenovirus containing GFP-ATF3 (ATF3) (Hanbio, Shanghai, China) was employed.

To ensure the specificity of ATF3–siRNA, rescue experiments were performed. After co-transfection of ATF3 with ATF3–siRNA, Western blotting was used to evaluating the ATF3 protein expression.

### Real-time quantitative-PCR

The total RNA was extracted from cultured podocytes using Trizol reagent (Invitrogen) according to the supplier’s protocols. Reverse transcribed into complementary DNAs (cDNAs) used the PrimerScript real-time reagent kit (Takara Biotechnology, Dalian, China), and then the cDNA was subjected to qPCR using Power SYBR Green PCR Master Mix (Takara Biotechnology, Dalian, China). The 2^−ΔΔCt^ method was used to quantify the relative expression levels of messenger RNA (mRNA). The primers used for qPCR are listed as follows: ATF3, forward 5′-GAGGATTTTGCTAACCTGACACC-3′, reverse 5′-TTGACGGTAACTGACTCCAGC-3′; NFATc1, forward 5′-CTCGGCCTTTGCCCATCTC-3, reverse 5′- AGGAGCACGGAGCATCTGA-3′; Bax, forward 5′-CTGGACCATAGGTCGGAGTG-3′, reverse 5′-AATTCGCCGGAGACACTCG-3′; Bcl-2, forward 5′-GTCGCTACCGTCGTGACTTC-3′, reverse 5′-CAGACATGCACCTACCCAGC-3′; and glyceraldehyde 3-phosphate dehydrogenase (GAPDH), forward 5′-AGGTCGGTGTGAACGG ATTTG-3′, reverse 5′-TGTAGACCATGTAGTTGAGGTCA-3′.

### Western blot

Protein extraction from the kidney cortex or cultured podocytes under different experimental conditions was conducted as previously described [22]. The nuclear protein is isolated and prepared as described in the Nuclear and Cytoplasmic Protein Extraction Kit (Nanjing KeyGEN Biotech, Nanjing, China). According to the manufacturer’s protocol, the protein concentration was evaluated using a protein assay reagent kit (Invitrogen, Thermo Fisher Scientific, Waltham, MA). Equal amount of proteins was separated on 9% sodium dodecyl sulfate–polyacrylamide gels and transferred onto polyvinylidene fluoride membranes (Millipore, Billerica, MA, USA). The membranes were blocked by 5% non-fat dry milk for 1 h at room temperature and then incubated overnight at 4 °C with the following primary antibodies: rabbit anti-ATF3 (Abcam, Cambridge, MA), rabbit anti-NFATc1(Abcam), rabbit anti-Histone (Cell Signaling Technology, Danvers, MA, USA), rabbit anti-Bax (Santa Cruz, Dallas, TX, USA), rabbit anti-Bcl-2 (Cell Signaling Technology), rabbit anti-GAPDH (Bioworld Technology, Nanjing, China), and rabbit anti-Histone (Cell Signaling Technology). Finally, membranes were detected using ECL Western Blotting Detection Reagents (Advansta, Menio Park, CA, USA).

### Flow cytometric analysis

After treatment, the podocytes were collected and incubated in an Annexin V-FITC/PI apoptosis detection kit (Nanjing KeyGEN Biotech, Nanjing, China) according to manufacturer’s protocol. Adenovirus containing GFP-ATF3 treated podocytes were in incubated in an Annexin V-APC/V450 apoptosis detection kit (BD Bioscience, Bedford, MA, USA). Briefly, podocytes were resuspended with 200 μl binding buffer and then incubated with 5 μl Annexin V (conjugated with FITC or APC) in the dark for 15 min. Then, the cells were stained with PI or V450 at room temperature for 15 min, followed by flow cytometric analysis using a FACScan flow cytometer and CellQuest software (BD).

### Immunofluorescent staining

Cultured podocytes planted on cover slides in six-well plates or frozen cryostat sections were subjected to immunofluorescence staining according to a standard immunofluorescence protocol described as previously [29]. The primary antibodies are listed as follows: goat anti-synaptopodin (Santa Cruz) and rabbit anti-ATF3 (Abcam). Secondary antibodies from Protein Tech Group, Inc. (FITC-donkey anti-goat IgG 488) and Cell Signaling Technology (Danvers, MA) (goat anti-rabbit Alexa Fluor 555) were used. All images were taken using laser confocal microscopy (LCSM, Zeiss KS 400, Postfach, Germany).

### ChIP–quantitative PCR assay

The chromatin immunoprecipitation assay was performed using the Thermo ChIP Kit (Invitrogen). Cells were cross-linked with 1% formaldehyde (final concentration) for 10 min at room temperature and terminated by adding glycine (1.25 M). After being washed twice using ice-cold PBS, cells were harvested in cell scraper and then resuspended with lysis buffer. The cell suspension was sonicated to fragments of 500 base pairs in length. The lysate was pre-cleared by incubation with protein G agarose and incubated overnight at 4 °C with either anti-ATF3 antibody (Santa Cruz Biotechnology) or non-immune IgG (Upstate Biotechnology, Inc.). To collect the immunoprecipitated complexes, protein G magnetic beads were incubated. After purified, DNA samples were amplified in a 7500 quantitative real-time PCR System (Applied Biosystems, Carlsbad, CA) and quantitated in triplicate by SYBR Green qPCR (Bio-Rad, CA, USA). The primer sequences that targeted mouse NFATc1 promoter are as follows: forward 5′TACAGCAAGCAATCCAGTTC 3 ′, reverse 5′ TCCCATCCCGCTAAATTACT3 ′. Data were analyzed using the 2^−△△CT^ method.

### Dual-luciferase reporter assay

NFATc1 promoter–luciferase reporter plasmids containing the NFATc1 promoter region were constructed by GeneCopoeia (Guangzhou, China). A dual luciferase reporter assay (SPDAD010, GeneCopoeia) was performed according to the manufacturer’s instructions. All experiments were repeated in triplicate.

## Statistical analysis

All values are expressed as means ± SEM. The statistical package SPSS for Windows ver. 19.0 (SPSS, Inc., Chicago, IL, USA) was used to statistical analysis. Multiple comparisons among the groups were performed using one-way ANOVA followed by Bonferroni adjustment/Tukey’s test or the Dunnett’s T3 test. Data from two groups were compared by Student’s *t* test. *P* value less than 0.05 was considered to be statistically significant.

## Results

### ATF3 is markedly upregulated in human and mouse proteinuric diseases

**Fig. 1:**
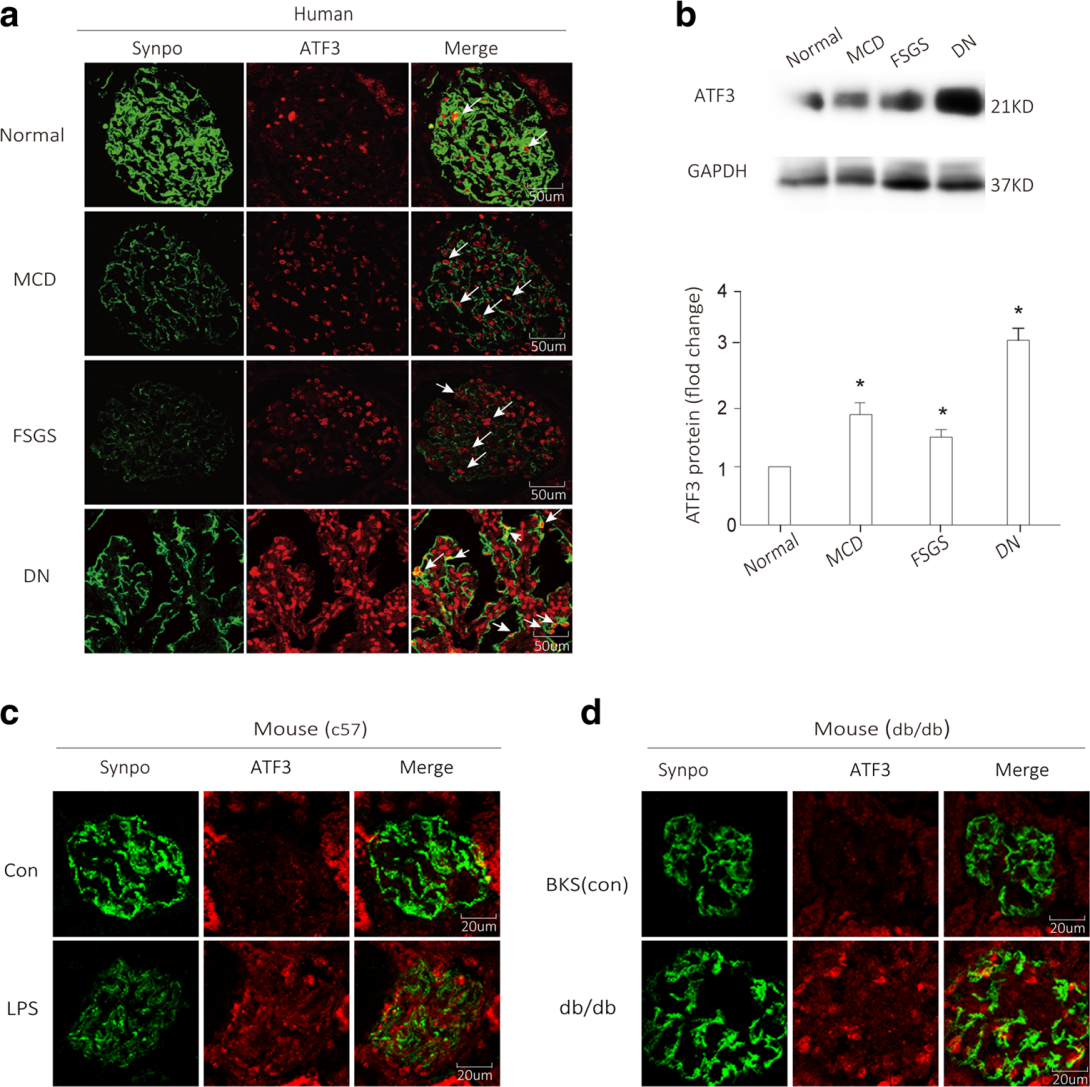
ATF3 is upregulated in podocytes in proteinuric patients and experimental proteinuric models. **a**, **b** ATF3 protein (red) is found in podocytes, as shown by double immunofluorescence with the podocyte marker synaptopodin (synpo, green) resulting in a partial yellow overlap. ATF3 expression is low in normal glomeruli from patients without glomerular disease. In contrast, ATF3 protein is increased in podocytes of proteinuric patients with minimal change disease (MCD, *n* = 4), focal segmental glomerulosclerosis (FSGS, *n* = 4), or diabetic nephropathy (DN, *n* = 6); similarly, western blotting showed an elevated level of ATF3 protein in patients with MCD, FSGS, or DN. **c**, **d** ATF3 is induced in podocytes in murine models of proteinuria. Compared with the control (C57 or BKS), ATF3 expression is elevated in the murine model of transient proteinuria (LPS-treated C57 mice) and the murine model of diabetic nephropathy (db/db mice) (*n* = 3) (color figure online)

ATF3, a 181-amino acid protein, is a member of the ATF/cAMP-response element-binding protein family of transcription factors and is maintained at low levels in quiescent cells [30, 31]. ATF3 was expressed in human glomerular cells (Fig. 1a), including podocytes, which were identified by synaptopodin labeling [32]. ATF3 was low in normal glomeruli from patients without glomerular disease. In contrast, ATF3 was increased in glomeruli from proteinuric patients with minimal change disease (MCD), focal segmental glomerulosclerosis (FSGS), and diabetic nephropathy (DN). To confirm the change of ATF3 expression in human proteinuric patients, we performed western blotting with kidney biopsy samples. We found low-level ATF3 protein in individuals without glomerular disease (Fig. 1b); in contrast, patients with MCD, FSGS, or DN had a significant increase in ATF3 protein, and an even stronger ATF3 expression was found in glomeruli cells from DN patients (Fig. 1b).

To further test which glomerular cells have increased ATF3 expression in animal models, we examined the localization of ATF3 within the glomeruli in animal models of proteinuria, including the LPS model, a well-recognized proteinuric mouse model [33], and the db/db mouse of diabetic kidney disease [34, 35]. We found low-level ATF3 expression in glomeruli from control mice (PBS treated C57BL/6 mice or non-diabetic BKS mice) (Fig. 1c, d); in contrast, ATF3 in all proteinuric models was substantially increased in glomerular cells, including podocytes (Fig. 1c, d).

### ATF3 was increased in HG-treated, LPS-treated, or Ionomycin-treated podocytes in vitro

**Fig. 2:**
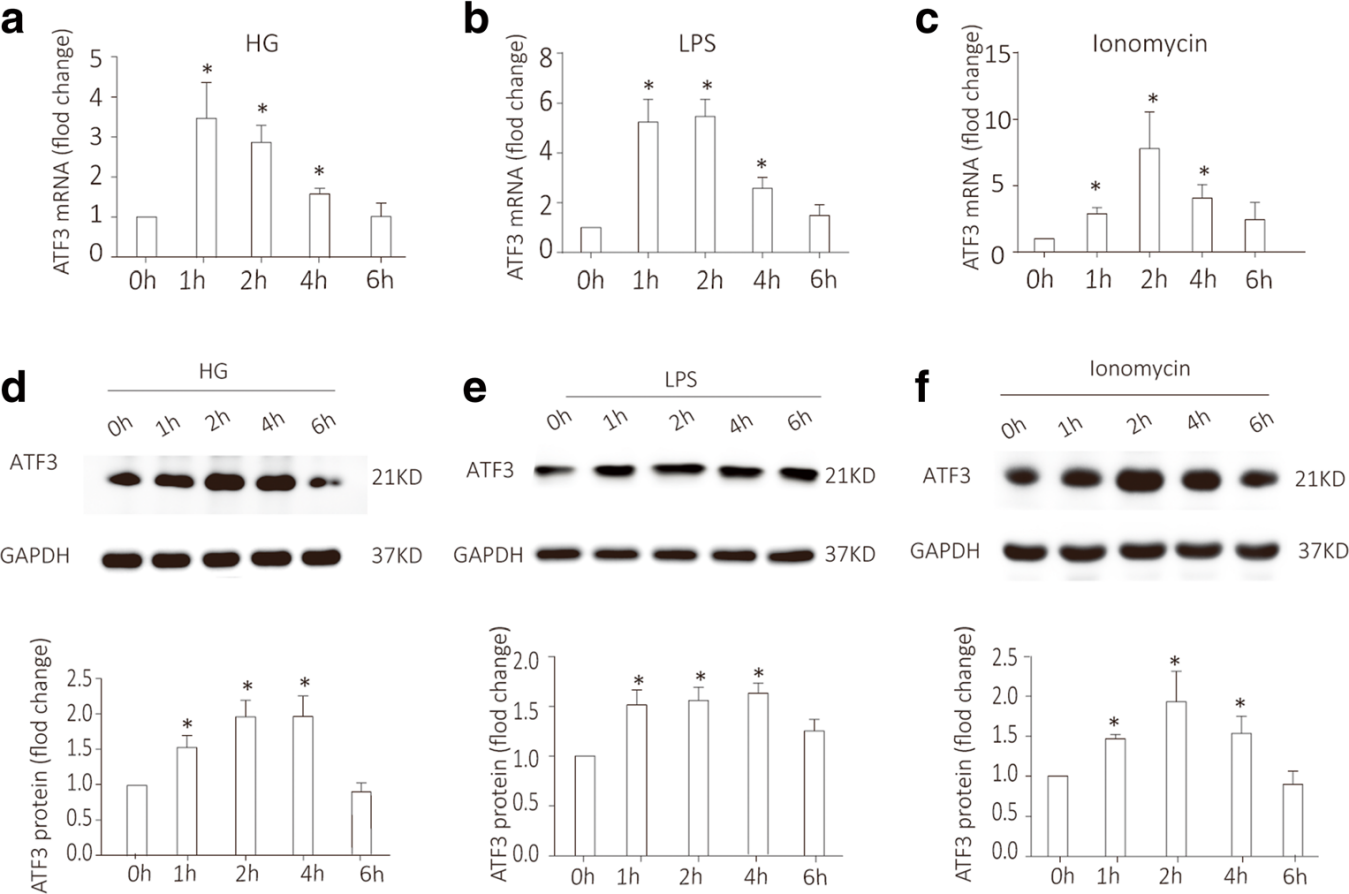
ATF3 was increased in high glucose (HG)-treated, lipopolysaccharide (LPS)-treated, or ionomycin-treated podocytes in vitro. **a**–**c** ATF3 mRNA is increased in high glucose (HG), lipopolysaccharide (LPS), or Ionomycin-treated podocytes for 1, 2, and 4 h (*n* = 4). **d**–**f** Similarly, ATF3 protein was increased in HG, LPS, or Ionomycin-treated podocytes for 1, 2, 4 h (*n* = 4). Data were from at least three independent experiments. *P < 0.05 versus controls.

We next used cultured differentiated podocytes, which express all known podocyte proteins including podocin [36]. To further confirm the change of ATF3 in injured podocyte, we treated the cultured podocytes with high glucose (HG), lipopolysaccharide (LPS), or Ionomycin for 1, 2, 4, or 6 h, respectively. As shown by qRT-PCR and western blotting, ATF3 mRNA (Fig. 2a–c) and protein (Fig. 2d–f) were obviously increased in HG-treated, LPS-treated, or Ionomycin-treated podocytes. This indicated that whereas ATF3 was nearly undetectable in uninjured cells, there was a clear increase in injured podocytes.

### Overexpression of ATF3 increased in vitro podocyte apoptosis and decreased podocin expression

**Fig. 3:**
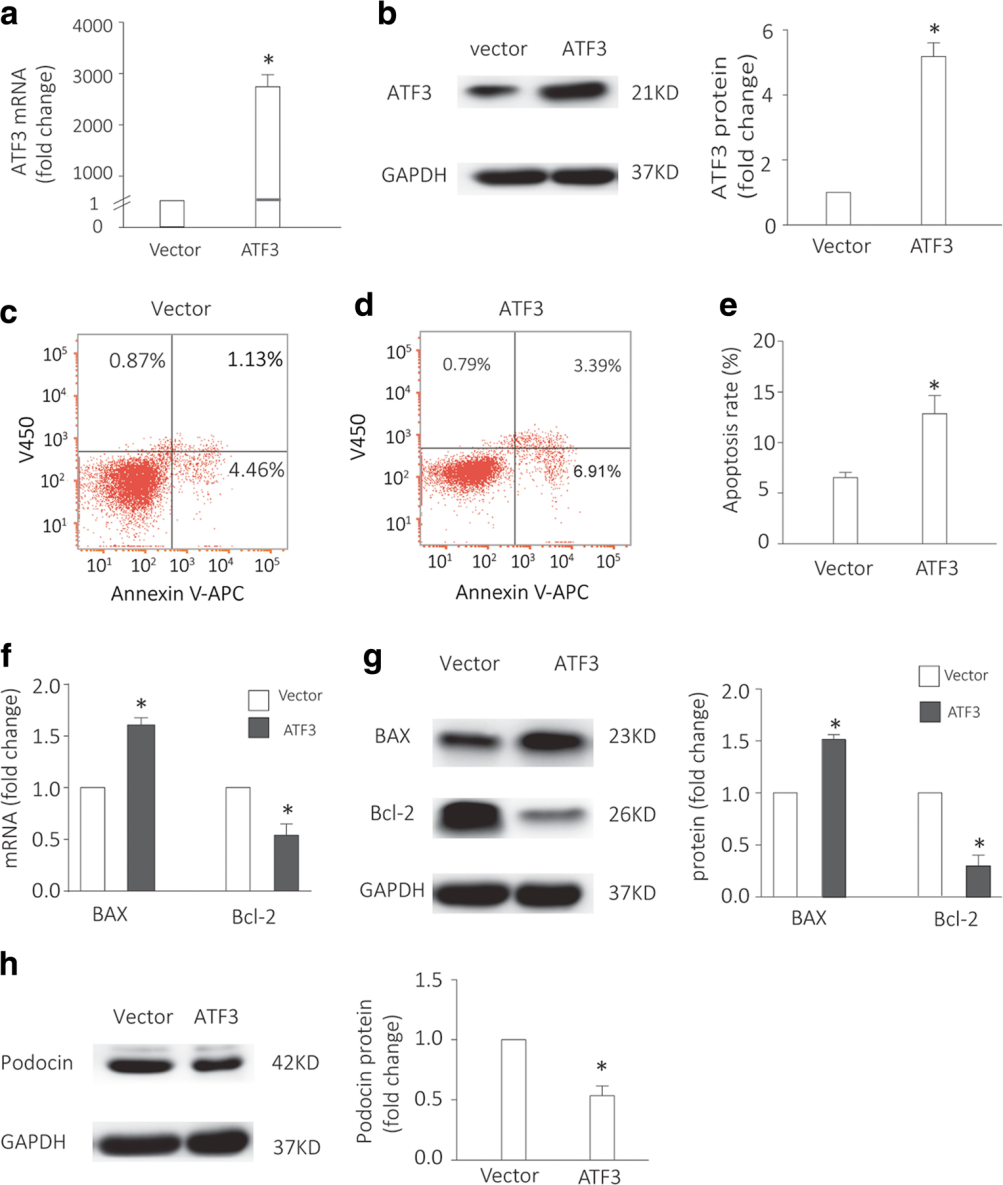
Overexpression of ATF3 increased podocyte apoptosis and decreased podocin expression. **a**, **b** Cell model of ATF3 overexpression in podocytes was established; ATF3 mRNA and protein expression were obviously increased in podocytes treated by adenovirus containing GFP-ATF3 (*n* = 3). **c**–**e** Flow cytometry analysis showed an elevated apoptosis in ATF3-overexpressed podocytes (*n* = 6). **f**, **g** In ATF3-overexpressed podocytes, the pro-apoptotic Bax mRNA and protein were increased while the anti-apoptotic Bcl-2 mRNA and protein were decreased (*n* = 4). **h** The expression of podocin, the podocyte marker, was reduced in ATF3-overexpressed podocytes (*n* = 3). Data were from at least three independent experiments. *P < 0.05 versus vector.

To explore the effect of ATF3 on podocyte, we established cell model of ATF3-overexpressed podocyte using adenovirus containing GFP-ATF3 (Fig. 3a, b). After treatment of adenovirus containing GFP-ATF3, we found a markedly increase of ATF3 mRNA and protein in treated podocytes. Since an enhanced loss of podocyte is always found in proteinuric patients, we investigated the effect of ATF3 overexpression on podocyte apoptosis, as well as Bax and Bcl-2, which are representative components in apoptosis [37, 38]. Flow cytometry analysis showed an elevated apoptosis in ATF3-overexpressed podocytes (Fig. 3c–e). Meanwhile, overexpression of ATF3 increased the pro-apoptotic Bax mRNA and protein and reduced the anti-apoptotic Bcl-2 mRNA and protein (Fig. 3f, g). To further confirm the effect of ATF3 on podocyte injury, we detected the expression of podocin, a key protein of podocyte slit diaphragm protein complex [39, 40]. We found that podocin protein was reduced in ATF3-overexpressed podocytes (Fig. 3h). Taken together, ATF3 overexpression increased podocyte apoptosis and injury.

### ATF3–siRNA knockdown reduced in vitro podocyte apoptosis and increased podocin expression

**Fig. 4:**
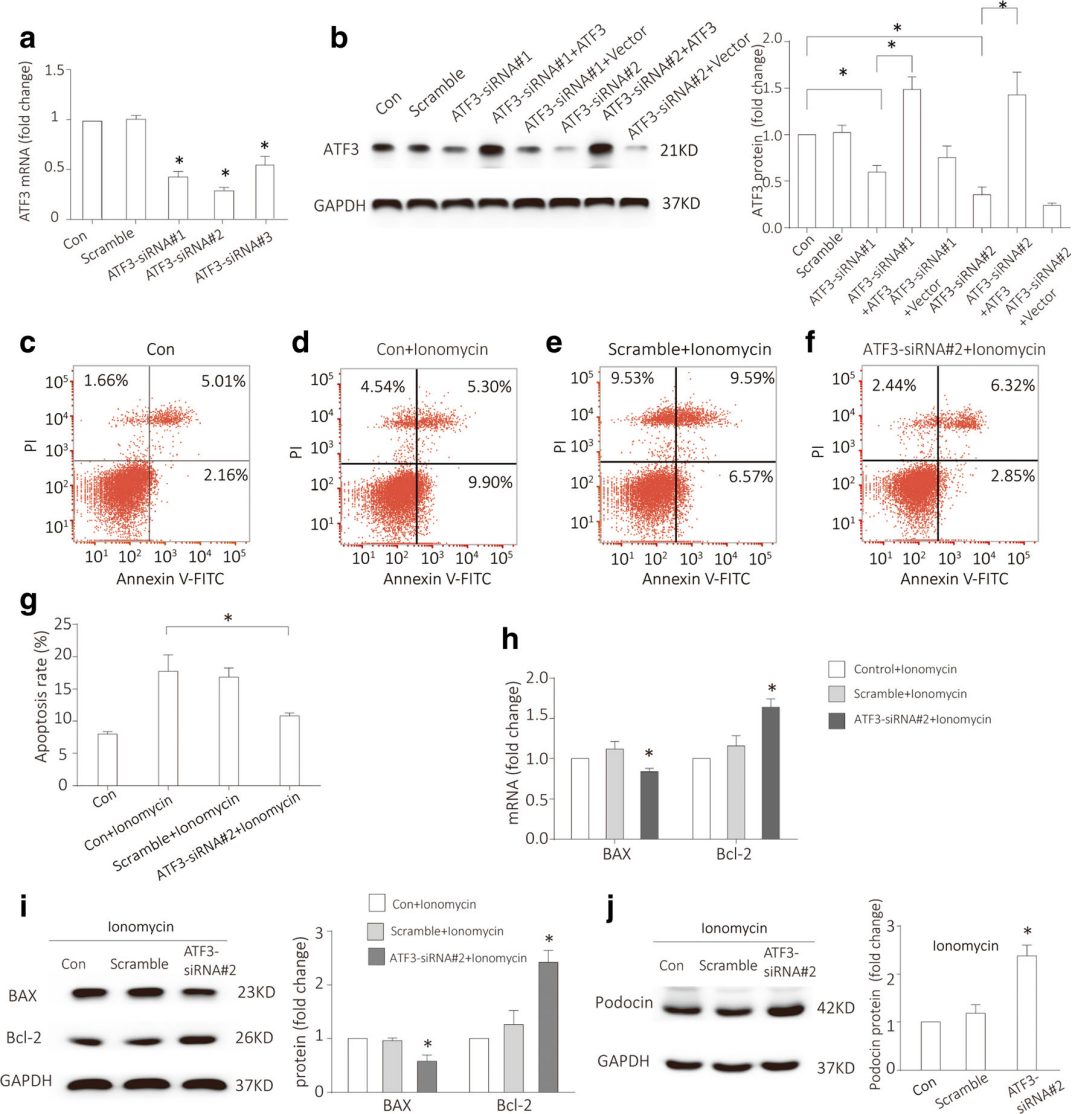
ATF3–siRNA knockdown reduced podocyte apoptosis and increased podocin expression. **a** ATF3 was silenced using ATF3–siRNA. Quantitative real-time RT-PCR analysis confirmed that the expression of ATF3 mRNA was knocked down by ATF3–siRNA (*n* = 4). b Rescue experiments of ATF3–siRNA. Western blotting showed that the expression of ATF3 protein was silenced down by ATF3–siRNA and the knockdown effect can be rescued by ATF3 (*n* = 3). **c**–**g** Podocytes were stained with Annexin V-FITC/PI for flow cytometry analysis. Apoptosis was significantly increased in Ionomycin-treated podocytes; in contrast, ATF3–siRNA knockdown reduced Ionomycin-induced podocyte apoptosis (*n* = 5). **h**, **i** In ATF3–siRNA knockdown podocytes, the pro-apoptotic Bax mRNA and protein were decreased while the anti-apoptotic Bcl-2 mRNA and protein were increased (*n* = 5). **j** The expression of podocin, the podocyte marker, was reduced in ATF3–siRNA knockdown podocytes (*n* = 3). Data were from at least three independent experiments. *P < 0.05 versus controls.

To explore the effect of ATF3 on podocyte, we established cell model of ATF3-silenced podocyte using ATF3–siRNA. Western blotting and quantitative real-time RT-PCR confirmed an efficient siRNA-mediated ATF3 gene silencing in podocytes (Fig. 4a, b). To confirm the specificity of ATF3 silence experiments, ATF3–siRNA rescue experiments were performed (Fig. 4b). After co-transfection of adenovirus containing ATF3 with ATF3–siRNA, we assessed ATF3 protein using Western blotting and found that the effect of ATF3–siRNA can be rescued by reexpressing ATF3. After treatment of Ionomycin, podocyte apoptosis was elevated; in contrast, ATF3–siRNA knockdown reduced apoptosis (Fig. 4c–g). And in ATF3–siRNA knockdown podocytes, the pro-apoptotic Bax mRNA and protein were decreased while the anti-apoptotic Bcl-2 mRNA and protein were increased (Fig. 4h, i). The expression of podocin, the podocyte marker, was reduced in ATF3–siRNA knockdown podocytes (Fig. 4j).

### The translocation of ATF3 to the nucleus was increased in injured podocyte

**Fig. 5:**
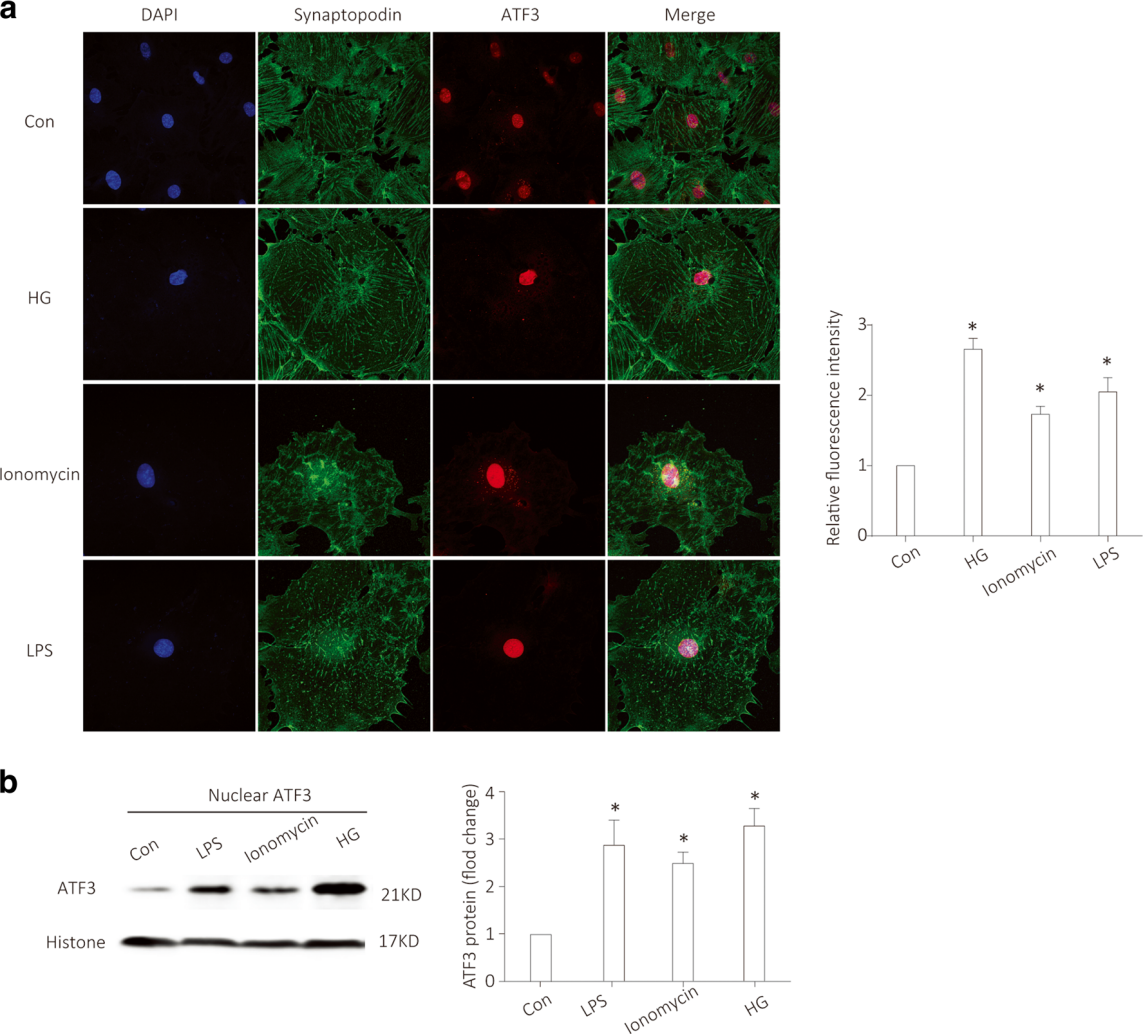
The translocation of ATF3 to the nucleus was increased in injured podocytes. **a** Nuclear ATF3 protein (red) was increased after 2 h-treatment of HG, Ionomycin or LPS in cultured podocytes, as shown by immunofluorescence with the podocyte marker synaptopodin (synpo, green) and DAPI-stained nuclei (blue) (*n* = 3). **b** After nuclear fractionation, nuclear protein is isolated and prepared. Western blotting showed an increased ATF3 protein in nuclei of podocytes treated by HG, Ionomycin, or LPS treated for 2 h (*n* = 4). Data were from at least three independent experiments. *P < 0.05 versus controls. (Color figure online)

ATF3 is a member of the ATF/cAMP-response element-binding protein family of transcription factors [30, 31]. To know if ATF3 can act as a transcription factor or not, we investigated the subcellular localization of ATF3 after injury. As shown by immunofluorescence (Fig. 5a), nuclear ATF3 protein was increased after 2 h-treatment of high glucose (HG), lipopolysaccharide (LPS), or Ionomycin, which were confirmed by western blotting (Fig. 5b), indicating that the translocation of ATF3 to the nucleus was induced in response to a variety of stress or injuries [31, 41].

### ATF3 directly modulates the regulation of NFATc1 gene promoter activity and then alters the transcriptional activity of direct targets of NFATc1 signaling (Wnt6 and Fzd9)

**Fig. 6:**
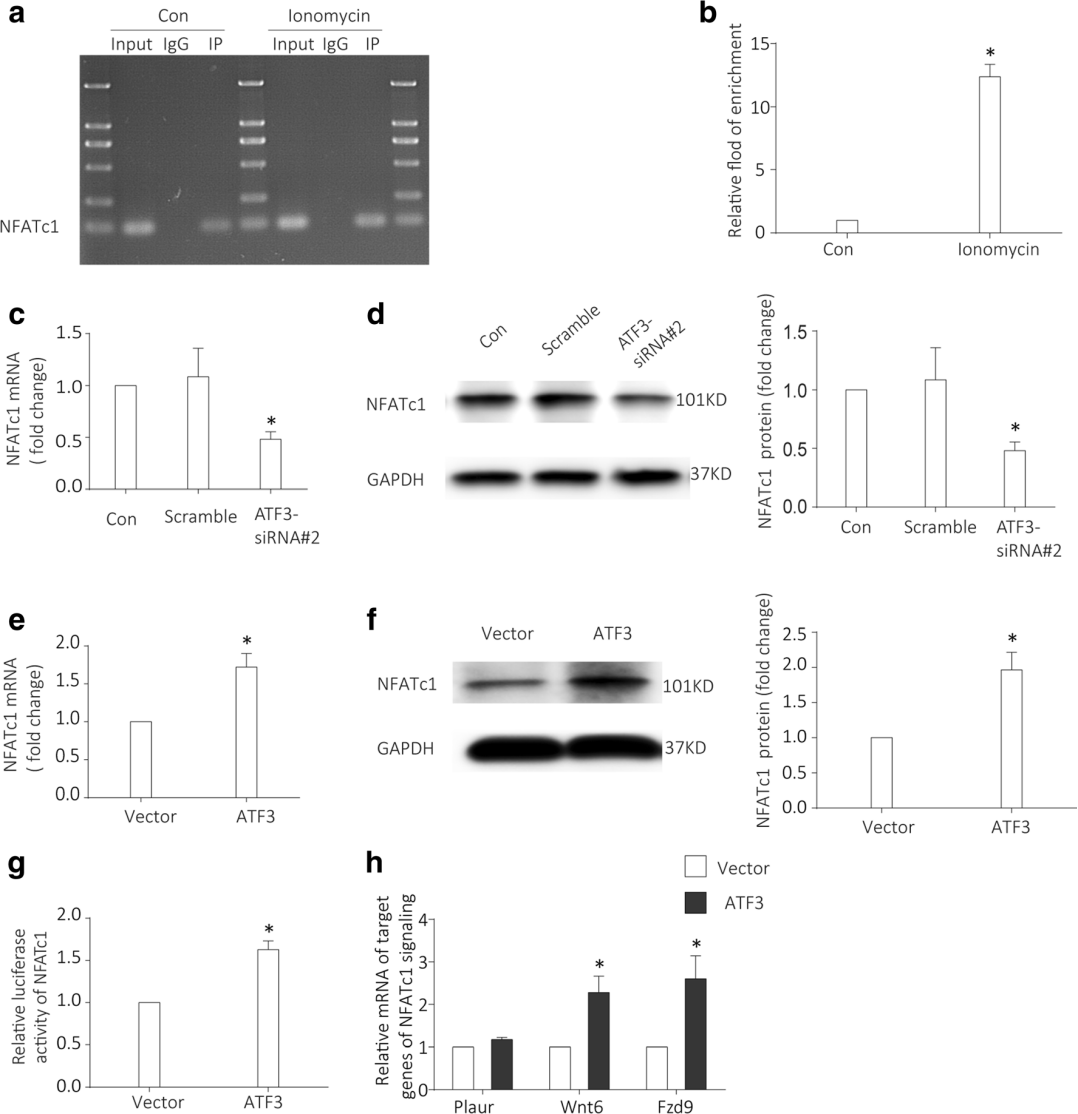
ATF3 directly modulates the regulation of NFATc1 gene promoter activity and alters the expression of Wnt6 and Fzd9, direct target genes of NFATc1 signaling. (**a**, **b**) A ChIP assay was performed to confirm the potential ATF3 binding site in the NFATc1 promoter region. IgG and input fractions were used as controls. ChIP analysis in podocytes using antibody to ATF3, followed by qPCR using the NFATc1 gene promoter-specific primer. The amplified promoter sequence is designed at the region 671–775 base pairs upstream of the transcription start site. DNA electrophoretogram showed that ATF3 binds to NFATc1 promoter (**a**), and ChIP–qPCR indicated that binding amount was increased in ionomycin-treated podocyte (**b**). Fold enrichment = [%(ChIP/Input)]/[%(Negative control/Input)](*n* = 3). **c**, **d** NFATc1 mRNA (**c**) (*n* = 3) and protein (**d**) (*n* = 6) were reduced in ATF3 knockdown podocytes. **e**, **f** In contrast, NAFTc1 mRNA (**e**) (*n* = 5) and protein expression (**f**) (*n* = 6) were elevated in ATF3-overexpressed podocytes. **g** The relative transcriptional activity of NFATc1 was elevated in ATF3-overexpressed podocytes treated by adenovirus containing GFP-ATF3. The secreted alkaline phosphatase (SEAP) luminescence was used as an internal control, and the ratio of Gaussian luciferase (Gluc) activity to SEAP was calculated for normalization. The relative transcriptional activity was converted into fold induction above the vehicle control value (*n*-fold) (*n* = 3). **h** Both Wnt6 and Fzd9, the direct transcriptional targets of NFATc1 signaling, were upregulated in ATF3-overexpressed podocytes (*n* = 4). The mRNA levels of the above genes were determined by qRT-PCR and standardized against GAPDH mRNA. All values are shown as the means ± SEM. *P < 0.05 versus controls

Because NFAT signaling mediates regulation of factors important for podocyte function and glomerulosclerosis [21, 23, 24], we explored whether ATF3 has a direct role in regulating NFATc1 gene promoter activity. A ChIP assay was performed to confirm the potential ATF3 binding site in the NFATc1 promoter region. The amplified promoter sequence is designed at the region 671–775 base pairs upstream of the transcription start site. DNA electrophoretogram showed that ATF3 binds to NFATc1 promoter (Fig.6a), and ChIP–qPCR indicated that binding amount was increased after administration of ionomycin (Fig. 6b). To test whether the expression of NFATc1 is altered in response to ATF3, we established cell models of ATF3-overexpressed podocyte and ATF3-silenced podocyte, respectively. ATF3–siRNA knockdown reduced NFATc1 mRNA and protein expression (Fig. 6c, d); in contrast, ATF3 overexpression increased NAFTc1 mRNA and protein expression (Fig. 6e, f). Meanwhile, we found that relative transcriptional activity of NFATc1 was elevated in response to ATF3 overexpression (Fig. 6g), indicating that ATF3 directly modulates NFATc1 gene promoter activity. Wnt6 and Fzd9 are the targets of NFAT signaling in podocytes and other systems and mediate podocyte injury as a result of NFAT activation [21, 42, 43]; we found that Wnt6 and Fzd9 were upregulated in ATF3-overexpressed podocytes, indicating that the upregulation may be a response to ATF3 overexpression.

## Discussion

**Fig. 7:**
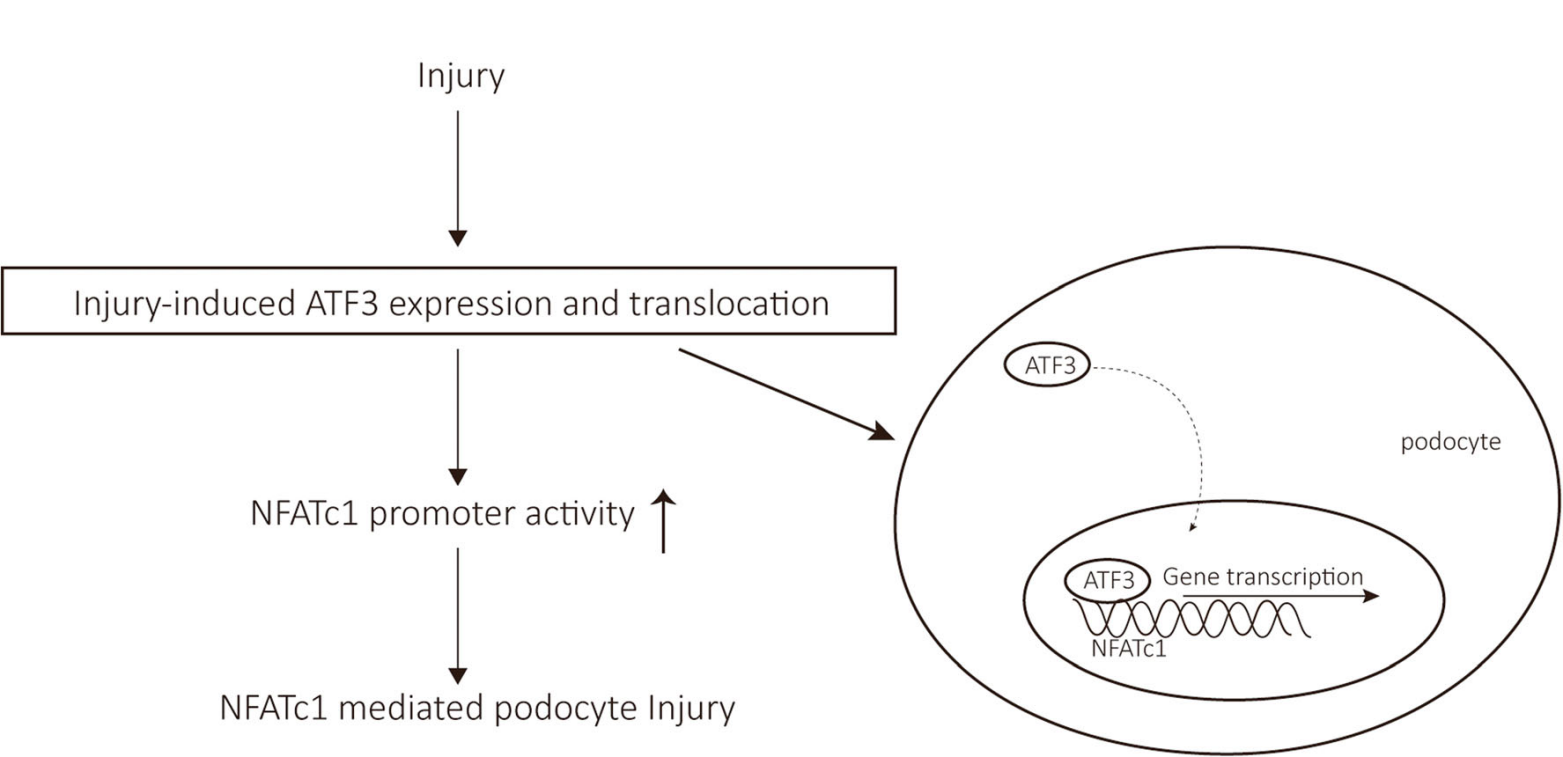
The inducible ATF3–NFAT axis aggravates podocyte injury

In this study, we found an inducible ATF3 expression in podocytes from the proteinuric patients. Overexpression of ATF3 aggravated in vitro podocyte injury and apoptosis; in contrast, inhibition of ATF3 induction prevented in vitro podocyte injury and apoptosis. Further molecular analyses demonstrated that ATF3 acted as a transcriptional activator in podocytes by upregulating NFATc1 gene promoter activity via direct binding to the NFAT promoter, indicating that the inducible ATF3–NFAT axis aggravates podocyte injury and apoptosis (Fig. 7).

The first finding is that there is an inducible ATF3 expression in podocytes from proteinuric patients with minimal change disease (MCD), focal segmental glomerulosclerosis (FSGS), and diabetic nephropathy (DN). ATF3 was previously recognized as an immediate early response gene induced by various stress signals [31]. However, this finding indicates an inducible ATF3 expression in podocytes under chronic conditions, but not under stress. The consequences of inducing ATF3 during non-stress responses in podocyte injury are not clear. The second finding showed that overexpression of ATF3 increased in vitro podocyte apoptosis and decreased podocin expression; in contrast, inhibition of ATF3 induction prevented in vitro podocyte injury and apoptosis. This result revealed the cellular pro-apoptotic and deleterious role of ATF3 during podocyte injury, which is consistent with other cells under stress conditions, such as stress-induced beta cell apoptosis [44], cell death, cell arrest, and apoptosis of fibroblasts [45]. However, ATF3 was also shown to execute its anti-apoptotic function in renal tubular epithelial cells under renal I/R injury [46].

The mechanism underlying the ATF3-mediated pro-apoptotic and deleterious effect during podocyte injury under chronic kidney disease is unclear. ATF3 may exert its effect by transcriptional control of target genes. In this study, we found an increased translocation of ATF3 to the nucleus upon stimulation. And ATF3 had a direct role in regulating NFATc1 gene promoter activity. In glomerular podocytes, ATF3 was shown to bind at the region 671–775 base pairs upstream of the transcription start site in the mouse NFATc1 promoter. Recently, multiple lines of in vivo evidence showed that activation of NFAT in podocytes causes proteinuria and glomerulosclerosis [21–24]. Podocyte-specific constitutively active NFAT mutant induces severe proteinuria, and the deleterious effect of a calcineurin/NFAT signaling pathway on podocytes and its pathogenic role in glomerular disease were demonstrated [21]. Meanwhile, a steady-state expression of TRPC6, a known pathogenic factor of glomerulosclerosis, is increased through a pathway dependent on NFATc1 [23]. In addition, 11R-VIVIT (a NFAT inhibitor) has been shown to reduce proteinuria and protect podocytes in diabetic db/db mice [29].

NFAT is involved in stimulating transcription of inducible target genes in immune and non-immune cells. Wnt6 and Fzd9 as the targets of NFAT signaling are detected in podocytes [21] and other systems, which mediate podocyte injury as a result of NFAT activation [42, 43]. Similarly, we found that podocyte-specific constitutively active NFATc1 increased uPAR expression and identified podocyte uPAR as a downstream target of NFAT in the pathogenesis of glomerulosclerosis [22]. In our study, we found that wnt6 and Fzd9, but not uPAR, were upregulated in ATF3-overexpressed podocytes, indicating that the upregulation of wnt6 and Fzd9 may be a response to ATF3 overexpression and mediate podocyte injury as a result of ATF3–NFAT axis activation. Although activation of ATF3–NFAT axis causes podocyte injury, ATF3 exerts its effect through other unknown pathways; thus, additional studies are needed to further explore whether these unknown mechanisms of ATF3 are important for podocyte injury induced by chronic conditions.

ATF3 as a homodimer has been shown to act as a transcriptional repressor [26, 47]. In contract, ATF3 can also function as a transactivator when it is heterodimerized with other basic leucine zipper proteins, such as c-Jun or JunB protein [27]. In line with this suggestion, ATF3 possibly activates transcription as a heterodimer in podocyte injury; additional studies are needed to further explore whether the heteromeric complexity of ATF3 are important for the deleterious effect mediated by ATF3 in vivo.

In summary, this study revealed an inducible ATF3 expression in podocyte injury induced by chronic disease, including minimal change disease (MCD), focal segmental glomerulosclerosis (FSGS), and diabetic nephropathy (DN). Our data indicate that inducible ATF3–NFAT axis aggravated podocyte injury.
